# Non-physician and physician preceptors in Landscapes of Practice: a mixed-methods study exploring learning for 1^st^-year medical students in clinical experiences

**DOI:** 10.1080/10872981.2023.2166386

**Published:** 2023-01-15

**Authors:** Jed D. Gonzalo, Deanna Graaf, Daniel R. Wolpaw, Erik Lehman, Britta M. Thompson

**Affiliations:** a Penn State College of Medicine, Hershey, Pennsylvania, USA; b Department of Medicine, Penn State College of Medicine in Hershey, Pennsylvania, USA

**Keywords:** Communities of Practice, Health Systems Science, Landscapes of Practice, mixed-methods, precepting, qualitative methods, social determinants of health, systems-based practice, undergraduate medical education

## Abstract

Medical education has traditionally relied on physician educators. With expanding Health Systems Science competencies, non-physician healthcare providers are required. To investigate preceptor-role types, communication frequency, and importance of preceptors in value-added patient navigator roles (PN) and clinical preceptorships (CP). Using a mixed-methods approach, medical students participating in PN and CP during the first year of medical school (*n*=191) identified individuals with whom they communicated and communication frequency (1=never, 7=frequently), and importance of preceptors to work/education (1=not important, 7=extremely important; open-ended responses). Quantitative data were analyzed via repeated measures using a mixed-effects model and McNemar’s test; effect size was calculated via Cohen’s d or Cohen’s h; qualitative data was analyzed using thematic analysis. Comparing ratings for non-physicians to physician healthcare professionals in PN, communication frequency (5.54 vs 3.65; *p*<0.001, d=1.18), importance to work (5.77 vs 4.28, *p*<0.001, d=0.89) and education (5.02 vs 4.12, *p*<0.001; d=0.49) were higher for non-physician educators. Comparing ratings for non-physicians to physician healthcare professionals in CP, communication frequency (4.93 vs. 6.48, *p*<0.001, d=1.33), importance to work (5.12 vs 6.61 vs, *p*<0.001, d=1.29) and education (4.32 vs 6.55, *p*<0.001, d=1.89) were higher for physician educators. Qualitative analysis indicated that non-physician healthcare providers in PN focused on Health Systems Science concepts, including social determinants of health and healthcare delivery. In PN, students observed collaboration from the perspective of multiple providers. In CP, healthcare providers, mainly physicians, focused on physician-centric clinical skills and interprofessional collaboration from the physician’s perspective. Educational benefits of non-physician healthcare professionals related to Health Systems Science in work-based clinical settings – or Landscapes of Practice – can help students understand systems-based concepts such as social determinants of health, healthcare delivery systems, and interprofessional collaboration. Differences in the educational value of non-physician healthcare educators perceived by students should be further explored.

## Introduction

Undergraduate medical education (UME) is transforming to better align with evolving physician roles in new systems of care [1]. A key focus of this transition involves Health Systems Science (HSS) curricula, including concepts such as interprofessional collaboration, health disparities, social determinants of health, high-value care, population health, and systems thinking [2]. Early experiential student roles, also known as value-added clinical systems learning roles (e.g., patient navigators (PN), health coaches), are being developed to catalyze learning in HSS [3–5]. Learning in these roles requires that students receive educational instruction, clinical guidance, and assessment by clinical preceptors to ensure goals of the experience are met [6–8].

Traditional medical student experiences primarily consist of biomedically-focused clinical preceptorships (CP) [9,10]. This student-physician dyad model focuses on clinical skills, including history-taking, diagnosis and therapeutics of patient care [9,10]. However, there are several challenges to the exclusive use of this preceptorship model in UME. First, integration of students into practices with physician preceptors is often viewed as ‘work added,’ requiring time and resources [11,12]. With increasing focus on improving efficiency and productivity of care delivery, newer models of student preceptorships and education in clinical settings are needed [5,13]. Second, and perhaps more importantly, accrediting bodies (such as the Liaison Committee for Medical Education) and collaborative groups (such as Canmeds and the Interprofessional Education Collaborative) expect students to be integrated into interprofessional care teams to develop the knowledge and skills for collaborative care at an early stage [14,15].

Despite this need for students to develop competencies in HSS and ‘function collaboratively on healthcare teams that include health professionals from other disciplines as they provide coordinated services to patients,’ most interprofessional education activities are stand-alone large events rather than longitudinal, authentic work-based experiences [9,10]. The ideal goal is to immerse students into real world clinical environments – or the Communities and Landscapes of Practice – where care delivery occurs with all members of the interprofessional care team, to learn clinical and systems-based skills, including collaboration [16–18]. While research suggests medical students find that learning with interprofessional students is enjoyable, allows them to learn about other professions, and helps them value working in collaborative teams, there is a paucity of research regarding medical students learning from non-physician healthcare professionals, particularly in work-based experiences and the Communities and Landscapes of Practice where students can enhance their HSS skills. Prior work has identified several benefits of interprofessional mentorship for medical students, including improving interprofessional learning and assessment of learners’ clinical skills [19]. Non-physician healthcare professionals on the interprofessional care team have traditionally had different roles with varying levels of autonomy; therefore, it is important to more fully elucidate medical students’ perceptions of learning from these educators and to understand potential implications for interprofessional education.

In this study, we used a mixed methods approach to determine students’ identification of non-physician healthcare professionals and physicians that they worked with and their perception of the education provided by these educators in an authentic longitudinal interprofessional experience that focuses on health systems sciences and social determinants of health.

## Methods

### Setting and participants

We studied a value-added clinical systems learning role performed by all first-year medical students at Penn State College of Medicine (PSCOM). Students serve as a patient navigator (PN) longitudinally within one clinical site between September and May for a minimum of two sessions per month (~50–60 hours) during the first year. PN goals include working with interprofessional care team members to explore health disparities and social determinants of health through identifying patients’ barriers to care, helping patients navigate complex health systems to obtain required care, and assisting patients in achieving better outcomes. These goals aligned with our need to evaluate the program in regards to the quantity and quality of interprofessional mentorship and the learning of HSS concepts (the basis of this study). The PN role was part of a larger Health Systems Science course in the first year focusing on concepts of high-value care, care coordination, population and social determinants of health, and leadership. The PN role provided the opportunity to be immersed into the healthcare delivery system and interprofessional care team to learn HSS concepts. This patient navigation role began in 2014 [20,21]. In the 2014–15 academic year, 85 of 150 students were placed across 16 sites, and in the 2015–16 academic year, 144 of 150 students were placed across 36 sites; clinical sites were diverse, including the physical medicine and rehabilitation hospital, primary care clinics, hemodialysis units, hospital transitions of care teams. During the first year, students also complete a one-week clinical preceptorship (CP) in the Spring semester (~50 hours) within one primary-care clinic. CP goals align with clinical skills, including history-taking, performing a physical examination, and communicating with patients. The CP provided an important experience that provided unique comparison information for the PN results because both experiences: were similar in time (~50 hours); were designed for first year students; were both experienced by students; had a designated site preceptor responsible for teaching, advising, and evaluating the student and communicating with the medical school regarding student progress; mentors in both experiences received a similar short orientation focused on course goals and expectations. Both also occurred in a clinical team-based care setting to allow for both physician and non-physician healthcare professionals outside of the assigned preceptor could also provide education and guidance [7,14]. Only data from students that had participated in both experiences and had signed consent to use their data as part of a data registry were included.

### Study design and data collection

To evaluate students’ perception of the education provided by physicians and non-physician healthcare professionals in an authentic longitudinal interprofessional experience focusing on health disparities and social determinants of health, we performed an embedded, mixed-methods sequential explanatory study, which involved the collection and analysis of both quantitative and qualitative data [22]. We chose this method to acquire an enriched understanding of students’ perceptions, which would have been limited by quantitative data alone. At the end of the year, shortly after completion of both experiences, students completed a modified networking inventory (Appendix 1), which asked the student to identify the healthcare individuals with whom they interacted for both experiences, the individual’s role or position, and the frequency of communication with each individual (1–7; 1=never, 4=occasionally, 7=frequently). Students were also asked to rate the importance of each individual to their work as a student at the clinical site, and the importance of the individual to their education (1–7; 1=not at all important, 4=somewhat important, 7=extremely important). Students were asked to reflect on why the highest-rated individual in each experience was important to their work as a student at the site and to their education (qualitative component of study). Due to anticipated analytic challenges involved with the data obtained from asking students to reflect on all individuals in each experience, we decided to only ask the students to provide information about the highest-rated individual. Data used in this study was approved as part of a larger education research registry by the PSCOM Institutional Review Board (#STUDY00000123).

### Data analysis

#### Quantitative

Characteristics of individual roles between experiences are reported descriptively. For analysis purposes, individual roles were collapsed into healthcare provider roles (physician, nurse, midlevel provider, care navigator, social worker, or other). Given the repeated measures for each student, a linear mixed-effects model that accounted for the correlation between the multiple observations for each student was employed. This model was used to compare the mean number of individuals and healthcare provider roles identified for PN and CP. We used McNemar’s test to compare proportions of students for PN and CP who identified communicating with each healthcare provider role at the site. Finally, ratings of communication frequency, importance to work and importance to their education were averaged across each site and healthcare provider role (physician/other) for each student, and compared using a linear mixed-effects model with factors for site, role, and the interaction between them. Effect sizes were calculated using either Cohen’s d statistics for means, or Cohen’s h statistics for proportions; values are categorized as: 0.20-small effect size, 0.50-medium effect size, 0.80-large effect size. Statistical significance was set at 0.05, and all analyses were implemented using either SAS or Stata/IC-8.

To better understand student perceptions regarding the importance of various healthcare provider roles to their work at the site and to their education, we performed a thematic analysis of student responses associated with the role and site [23]. This allowed us to explore perceived educational benefit of non-physician healthcare provider and physician roles in both PN and CP. One investigator (D.G.) independently coded open-ended responses and held regular adjudication sessions with two co-investigators (J.G., B.T.) to allow for additional code creation, elimination, and refinement. Data saturation was achieved after analysis of 50 responses, but the entire dataset was coded. Finally, co-investigators discussed themes and representative quotations. We used data management support program NVivo 11 QSR International, Berlin.

## Results

Data from 191 students (response rate: 83.4%) who experienced both PN and CP during two academic years (2014-15 = 78 students, and 2015-16 = 113 students) was used. The total number of individuals identified by students in both experiences was 1178 (Table 1), for a total mean of 6.2 providers identified per student split evenly between PN and CP (PN mean = 3.1 providers, CP mean = 3.1 providers, *p* = 0.945). Some students identified individuals in their network that were the same healthcare provider role, such as indicating multiple physician providers with whom they communicated within a site.

**Table 1.** Professional Roles and Number of Educators in Patient Navigation and Clinical Preceptorships (*n* = 191 students).

Role	Patient Navigation	Clinical Preceptorship	Total
Physician	146	366	512
Nurse (RN, CRNA, LPN)	131	76	207
Social worker	92	1	93
Other	73	84	157
Care navigator (e.g., care coordinator)	66	1	67
Midlevel provider (e.g., physician assistant)	30	35	65
Care manager	21	1	23
Therapist (e.g., speech, physical)	12	1	13
Dietician/nutritionist	7	1	8
Pharmacist	6	16	22
Psychologist/counsellor	4	1	5
Translator	2	3	5
Dental hygienist or dentist	0	2	2
Total	590	588	1178

Note: Abbreviations: CRNA=certified registered nurse anesthetist; LPN=licensed practical nurse; RN=registered nurse

### Comparisons of different preceptor roles

To better understand the proportion of students identifying a specific healthcare provider role, we determined how many students included each type of healthcare provider role (i.e., physician, nurse, social worker, etc.). We found that students in PN identified more unique healthcare provider roles (mean = 2.43) compared with CP (mean = 1.93) (*p* < 0.001, d = 0.51). In PN, 61% of students identified at least one physician healthcare provider while all students in CP (100%) did the same (*p* < 0.001, Cohen’s h = 1.34). In PN, almost all students (93%) identified at least one non-physician health care provider while for CP, 57% reported the same (*p* < 0.001, Cohen’s h = 0.89). Table 1 provides a detailed breakdown of various health care providers.

### Frequency of communication, and importance of roles to work and education

Students in PN indicated that they communicated with non-physician healthcare providers (mean = 5.54) more frequently than physician providers (mean = 3.65) (*p* < 0.001, d = 1.18). For comparison, students in CP rated their frequency of communication with non-physician providers (mean = 4.93) as less than physician providers (mean = 6.48) (*p* < 0.001, d = 1.33) – see Table 2.

**Table 2.** Frequency of Communication and Importance of Educator Roles to Clinical Work and Education.

	Patient Navigation (mean, response 1–7)		Clinical Preceptorship (mean, response 1–7)			
Item^a^	Non-physician healthcare provider	Physician healthcare provider	Non-physician healthcare provider	Physician healthcare provider		p-value
Communication frequency	5.54	3.65	4.93		6.48	<0.001
Importance to clinical work	5.77	4.28	5.12		6.61	<0.001
Importance to education	5.02	4.12	4.32		6.55	<0.001

^a^Items included the following answer choices: communication (1=never, 4=occasionally, 7=frequently) and importance to clinical work/education (1=not at all important, 4=somewhat important, 7=extremely important).

Students in PN indicated that non-physician providers were significantly more important to their work at the site (mean = 5.77) than physician providers (mean = 4.28) (*p* < 0.001, d = 0.89). For comparison purposes, students in CP indicated that non-physician providers were less important to their work at the site (mean = 5.12) compared to physician (mean = 6.61) (*p* < 0.001, d = 1.29). Students in PN indicated that non-physician providers provided more educational value (mean = 5.02) than physician healthcare providers (mean = 4.12) (*p* < 0.001, d = 0.49). For comparison purposes, students in CP rated the educational value of non-physicians (mean = 4.32) as significantly less valuable than physician providers (mean = 6.55) (*p* < 0.001; d = 1.82).

### Importance of preceptors to education and work

We identified five themes related to the benefits of working with and learning from healthcare providers across both PN and CP experiences. A total of 491 coding references were categorized by theme, healthcare provider type (physician/non-physician), and clinical experience (PN/CP). In PN, the most frequent theme associated with non-physician providers was social determinants of health and the least was clinical skills. The most frequent theme associated with physician providers in PN was clinical skills and the least was humanism. For comparison purposes, in CP, the most frequent theme associated with non-physician providers was interprofessional collaboration while the least was social determinants of health and healthcare delivery. The most frequent theme associated with physician providers in CP was clinical skills while the least was humanism and healthcare delivery system. Table 3 includes the coding reference counts and representative quotations for each of the following themes.

**Table 3.** Qualitative Analysis with Coding References of Educational Benefit of Physicians and Other Health Care Providers in Clinical Preceptorships and Patient Navigation.

Themes	Coding Reference Counts			Representative Quotations
	Clinical Experience	Non- physician providers	Physician Providers	
Clinical Skills *n* = 213	PN (48)	27	21	[Nurse] ‘I had limited clinical experience upon starting patient navigation and had many reservations about how to handle patient interactions and how I might respond to certain scenarios. However, my trepidation was met with open arms and I was guided throughout the process. The nurse showed extreme kindness to patients and took the time to offer patient education for new patients and patients with a history of Inflammatory Bowel Disease. Their patience and patient-centered focus was a priority integrated into each encounter.’
	CP (165)	10	155	[Physician] ‘Dr. X determined the tasks I would complete for each patient, and helped me improve my style of presenting patients to physicians and my style of writing SOAP notes. She helped me refine my clinical skills by pushing me to identify murmurs and lung sounds. She modeled excellent physician-patient relationships, and also introduced me to underserved medicine.’
Collaboration*n* = 103	PN (45)	37	8	[Nurse case manager] ‘[She] helped [us] set up agendas for home visits, gave us patient lists for phone calls and acted as a mentor for all advice regarding how to best manage patients. She was our “go between” for contacting patients and working with the rest of the health care team. Her role was essential in ensuring my partner and I were making significant contributions to the clinic. I got to see the entire healthcare team work with an ALS patient.’
	CP (58)	33	25	[Nurse director] ‘She helped my partner and I set up agendas for home visits, gave us patient lists for phone calls and acted as a mentor for all advice regarding how to best manage a patient. She was our go between for contacting patients and working with the rest of the healthcare team. Her role was essential in ensuring my partner and I were making significant contributions to the clinic. All the roles in the clinic were important to my education, especially when I observed the team work with ALS patients and how they explained the disease to the patients. I got to see the entire healthcare team work with an ALS patient.’
Social Determinants of Health n = 82	PN (72)	69	13	[Nurse] ‘The main coordinators of care at [the] Mission who know the men who live there the best were the ones who set up our patients for the day and served as our main resource for information and questions regarding how to help [them]. They led us to resources for our patients and taught us about medications and services the patients had.’
	CP (10)	0	10	[Physician] ‘He was the person I shadowed and I learned a lot about clinical diagnosis from him. He also helped me realize a lot of the difficulties in navigating healthcare that most patients experience because many of his patients had a lower income.’
Health Care Delivery System n = 54	PN (49)	39	10	[Care coordinator] “They provided me with education on current payment systems and barriers to access. I made a positive impact on my patients’ lives and it was largely due to [the care coordinator’s] guidance. They educated me regarding various topics of our current health care system, current payment systems, the details of Medicaid and Medicare, and barriers to access. Most of all, their passion for helping patients created a positive work environment that made me excited to learn.”
	CP (5)	0	5	[Physician] ‘He provided both the background and data necessary for our operations. We discussed several global issues facing the health system, especially related to the Medicare population in a primary care setting. Because of his experience in population health and policy, [Dr. X] was offered ample information, which helped me more concretely understand implications of problems with health care.’
Humanism *n* = 39	PN (33)	30	3	[Nurse] ‘Our advisor devised an entire program for us. She worked closely with us and made sure we understood our patients, their problems, and how a simple phone call could change someone’s life – so we also were motivated to do our job. We had very clear education about mental health and it was essential for helping us do the job. The nurses made us understand how important it is to listen to patients and really practiced what they preached. It was great to see them in action and be encouraged that all of our communications/feelings stuff will be beneficial in the future.’
	CP (6)	3	3	[Physician] “Dr. X has always been a wonderful role model for how to treat patients and assess problems. She was supportive and organized in running the X clinic, while ensuring students were involved and included. She has shown me how to problem solve and treat patients with respect. I watched her interactions with patients – she always spoke to patients directly, offering her chair to patients’ families, squatting on the floor during visits.”

**Social Determinants of Health**. Both experiences allowed students to develop an enhanced understanding of the factors influencing patient outcomes and medical care (e.g., financial resources, transportation), availability of resources, and how to locate community resources. However, in PN, students learned about barriers to care and actively worked with patients to pursue solutions and identify community resources, especially when working with non-physician healthcare providers.

**Health Care Delivery System**. In PN, students indicated that providers, especially non-physicians, discussed a range of HSS concepts, including health system data and billing systems, community preventive measures for reducing healthcare costs, healthcare payment system and insurance (e.g., Medicaid), logistics and flow of the health system, gaps in care, and the effect of inefficiencies in health care on patients.

**Clinical Skills**. In CP, this code represented the majority of codes associated mainly with physician healthcare providers. Students reported enhancing their clinical skills, including physical diagnosis, history-taking and interviewing, communication, and clinical decision making. In PN, students reported learning clinical skills, including disease processes, interpretation of lab tests and results, shadowing procedures and surgeries, navigating the electronic health record (EHR), and communicating with other care providers on care teams.

**Interprofessional Collaboration**. In PN, students learned about communication and collaboration between healthcare professionals within and across the health system as well as the patient role within care teams, mainly from non-physician healthcare providers. In CP, students witnessed the interactions and leadership of physicians with non-physicians (i.e., providing constructive feedback and collaborating with the team to ensure efficient workflow).

**Patient-Centered Care and Humanism**. Students felt that working with non-physician healthcare providers in PN allowed them to observe compassion and empathy and efforts to understand a patient’s background. Few students identified this theme for physician healthcare providers in PN or CP.

## Discussion

The purpose of this study was to determine the non-physician healthcare professionals and physicians that students worked with in a value-added, authentic longitudinal systems role (patient navigation, or PN) and their perception of the education provided by these educators. Within PN, students reported communicating with non-physician providers more frequently than physicians, indicated that non-physician providers were more important to their work at the site, and that non-physician providers were somewhat more important to their education. These findings align with the objectives of the PN program to create an environment where students can communicate with and learn from interprofessional healthcare clinicians.

Qualitative analysis indicated that non-physician healthcare providers in PN focused on Health Systems Science (HSS) concepts, including a large focus on social determinants of health, healthcare delivery, and humanism. In PN, students observed collaboration from the perspective of multiple healthcare providers. In CP, physician healthcare providers focused on physician-centric clinical skills as well as interprofessional collaboration from the physician’s perspective. These data suggest that non-physician healthcare providers working in authentic experiences such as PN can educate to improve students’ knowledge and skills in areas such as social determinants of health with multiple members of health care teams [10,16–18]. While CP-type experiences are vitally important for students to learn clinical skills, our data suggest that these experiences may limit students’ interactions with non-physician members of the team. We suggest that if students are not provided the opportunity to experience value-added authentic systems roles, they potentially may be at a disadvantage to learn and apply concepts of social determinants of health, healthcare delivery systems, and interprofessional collaboration [24].

In US medical schools, the Liaison Committee on Medical Education (LCME) requires interprofessional education (IPE) as a requirement for medical student experiences, and many resources are being allocated to these programs. In recent years, IPE-related experiences are blending classroom-based experiences with collaborative care settings, i.e., at the ‘nexus’ of education and clinical practice where both join forces for sustainable change [25]. Over the course of their clinical experiences, medical students encounter many Communities of Practice featuring a range of healthcare professionals [16,17]. The Landscapes of Practice framework is a particularly helpful way to conceptualize this interprofessional journey. Ideally, students are not limited to only observing these various clinical settings, but are afforded opportunities to learn, engage, and contribute (‘sojourn’) [18]. These cross-boundary encounters across different professional teams allow them to become ‘knowledgeable’ in a range of Communities of Practice across the healthcare landscape [18,26]. However, most current medical student clinical experiences are not designed to take advantage of the learning opportunities across this diverse landscape, but rather to advance doctoring skills. This tends to make the experiences predominantly physician-centric and limits meaningful engagement and perspective-taking with other team members [27]. There has been increasing attention to this gap, with recommendations to either enhance current activities or develop new experiences for interprofessional engagement in the process of providing care [5,10,13,28,29]. We propose that both PN- and CP-type experiences are unique and provide important opportunities for students and healthcare providers. Our findings highlight the potential to create authentic, longitudinal experiences with non-physician healthcare providers that provide unique opportunities for medical students to learn and apply health systems science concepts, such as social determinants of health and health disparities. We believe these learning relationships are particularly important in professional development during formative years of training [30].

While our results were positive, our effect size calculations also revealed an especially large gap in students’ perceived educational value of non-physician healthcare providers within the clinical setting compared to that of physicians. We submit this may be due to 1) limited training of non-physician healthcare educators or 2) ‘hierarchies of education.’ [27] In PN, many non-physician healthcare providers had never been asked to precept medical students – this program was their first experience in this role. In medical schools across the country, although they may occasionally supervise students, faculty with degrees in nursing, public health, management, or social work (i.e., interprofessional faculty) have not been identified as core faculty [8,9,31]. As emphasis on HSS, including social determinants of health and healthcare delivery increases in both health care and medical education, so will the need to provide faculty development and help the non-physician healthcare providers to gain competencies in teaching [31–33]. We believe it is the responsibility of medical schools and affiliated Academic Health Centers to embrace, validate, and elevate non-physician healthcare providers by revisiting the concept of faculty status, compensation, and continuing professional development for these educators.

Just as importantly, we must help medical students see the value of learning from non-physician healthcare professionals. Our results show that students may value physicians more than non-physician healthcare providers with respect to their educational priorities, highlighting an opportunity to articulate to students the potential benefits of interprofessional mentorship. It may be that students enter these practice-based experiences influenced by traditional medical education hierarchies that prioritize learning from physicians or other academic faculty. It is likely worth exploring this point further, however, as potential professional growth opportunities with non-physician faculty extend to critical areas well beyond medical knowledge and clinical skills. These interprofessional faculty have a deep tradition in patient-centered orientation and skills, connecting with the narratives of patient’s lives, families, and communities. They can serve not only as role models, but also as important triggers for students to challenge their perspectives and expand their mental models.

Professional identity formation is a critical component of physician education in 21^st^-century medicine [34]. Traditionally, clinical experiences have relied upon physician preceptors to teach students core ‘doctoring skills,’ as reflected in our results in CP. There is tremendous value to these experiences as they expose students to physician-based skills and roles within health systems. However, sole use of these experiences may limit a student’s view of the health system, potentially reinforcing a physician-centric view of care delivery models. This is at odds with the transforming healthcare landscape and the increasing emphasis on a professional identity that prioritizes effective collaboration, systems thinking, and a broader understanding of the determinants of health and the impact of health care delivery systems on patient outcomes [1,4,14,35–38]. This transition requires student physicians to develop a working knowledge of non-physician providers and caregivers, their roles, and the contributions they can make in new models of care delivery. It also requires students to increase their awareness of the available resources for patients, communities, and populations. Students’ professional identity formation is much more likely to be shaped by preceptors and role models in workplace ‘Landscape of Practice’ settings than through classroom-based instructional methods [10,16–18,26,39–42]. Notably, the least identified learning theme in both CP and PN was ‘humanism.’ Considering an increasing focus on humanism as a critical component of professional identity formation, this finding merits further exploration in all types of mentored clinical experiences. To our knowledge, this is the first study to investigate the role of interprofessional educators in the clinical experience of first-year medical students and contrast it to traditional physician-centric clinical placements.

Several limitations should be acknowledged in this study. Although CP is commonly used in US medical schools, PN is not widely adopted which may limit the transferability of these findings to different settings [5]. We believe, however, our study is a starting point for exploring the impact of non-physician healthcare providers in helping students navigate the site as well as educate students in important areas such as social determinants of health and health systems sciences.

In conclusion, our results support the development of innovative clinical and health systems experiential roles for early medical students that feature important roles for both physicians and non-physician healthcare providers. Working with non-physician providers in work-based clinical settings expands opportunities for the applied learning of HSS concepts such as social determinants of health, healthcare systems, and interprofessional collaboration. We believe these experiences have unique potential for supporting medical student professional identity formation as collaborative and knowledgeable health care providers. It is our hope that this study will catalyze an ongoing national discussion on the role of non-physician healthcare providers in medical education.

## Appendix 1

**Data Collection Instrument**

Students were asked to complete this instrument for both clinical preceptorships and patient navigation.

### Educational Preceptorship Instrument

Section 1: Please refer to your **patient navigator experiences (other grid – Clinical Preceptorship**) over the past year when completing the following grid. Please identify all healthcare providers with whom you communicated (in any form). Please remember to consider all methods of communication (face-to-face, phone, reading what was written by the person in this role, etc.)

**Table:**

Role	Person (indicate position or role, for instance, physician)	On an average week that you were at the site, how often did you communicate with this individual? 1 = Never 2 3 4 = Occasionally 5 6 7 = Frequently	How important was this individual to your work at the site? 1 = Not at all important 2 3 4 = Somewhat Important 5 6 7 = Extremely Important	How important was this individual to your education? 1 = Not At All Important 2 3 4 = Somewhat Important 5 6 7 = Extremely Important
1				
2				
3				
4				
5				
6				
7				
8				
9				
10				

In relation to the grid you just completed above:

1. For the person you rated as most important to your work at the site, describe why they were important.
2. For the person you rated as most important to your education, describe why they were important.

## References

[1] Lucey CR. Medical education: part of the problem and part of the solution. JAMA Intern Med. 2013;173(17):1639–10. 23857567 10.1001/jamainternmed.2013.9074

[2] Gonzalo JD, Dekhtyar M, Starr SR, et al. Health systems science curricula in undergraduate medical education: identifying and defining a potential curricular framework. Acad Med. 2017;92(1):123–131. DOI:10.1097/ACM.0000000000001177 27049541

[3] DG GJ, Kass LE, Promes SB, et al. Medical students as systems ethnographers: exploring patient experiences and systems vulnerabilities in the emergency department. Academic Emergency Medicine Education and Training. 2017;1(3):225–233. 30051039 10.1002/aet2.10038 PMC6001711

[4] Gonzalo JD, Haidet P, Papp KK, et al. Educating for the 21st-century health care system: an interdependent framework of basic, clinical, and systems sciences. Acad Med. 2017;92(1):35–39. DOI:10.1097/ACM.0000000000000951 26488568

[5] Gonzalo JD, Dekhtyar M, Hawkins RE, et al. How can medical students add value? Identifying roles, barriers, and strategies to advance the value of undergraduate medical education to patient. Care and the Health System Acad Med. 2017;92(9):1294–1301. 10.1097/ACM.0000000000001662 28353500

[6] Billay D, Myrick F. Preceptorship: an integrative review of the literature. Nurse Educ Pract. 2008;8(4):258–266. 17988946 10.1016/j.nepr.2007.09.005

[7] Yonge O, Billay D, Myrick F, et al. Preceptorship and mentorship: not merely a matter of semantics. Int J Nurs Educ Scholarsh. 2007;4(1):Article19. 18052917 10.2202/1548-923X.1384

[8] Gonzalo JD, Chang A, Wolpaw DR. New educator roles for health systems science: implications of new physician competencies for U.S. medical school faculty. Acad Med. 2018;94(4):501–506. 10.1097/ACM.0000000000002552 30520810

[9] Ludmerer KM. Time to heal : American medical education from the turn of the century to the era of managed care. Oxford; New York: Oxford University Press; 1999.

[10] Gonzalo JD, Thompson BM, Haidet P, et al. A constructive reframing of student roles and systems learning in medical education using a communities of practice lens. Acad Med. 2017;92(12):1687–1694. 28640036 10.1097/ACM.0000000000001778

[11] Grumbach K, Lucey CR, Johnston SC. Transforming from centers of learning to learning health systems: the challenge for academic health centers. JAMA. 2014;311(11):1109–1110. 24643597 10.1001/jama.2014.705

[12] Lin SY, Schillinger E, Irby DM. Value-added medical education: engaging future doctors to transform health care delivery today. J Gen Intern Med. 2015;30(2):150–151. 25217209 10.1007/s11606-014-3018-3 PMC4314499

[13] Gonzalo JD, Hammoud MM, Schneider GW. Value-added roles for medical students. Elsevier. 2022;1:2–28.

[14] Interprofessional Education Collaborative. Core competencies for interprofessional collaborative practice: report of an expert panel. 2012. https://www.aamc.org/download/186750/data/core_competencies.pdf. Accessed February 29, 2013.

[15] Liaison committee for medical education. http://www.lcme.org/.

[16] Wenger E. Communities of practice: learning, meaning, and identity. Cambridge, U.K.; New York, N.Y: Cambridge University Press; 1998.

[17] Mann KV. Theoretical perspectives in medical education: past experience and future possibilities. Med Educ. 2011;45(1):60–68. 21155869 10.1111/j.1365-2923.2010.03757.x

[18] Wenger E, Fenton-O’Creevy M, Hutchinson S, et al. Learning in landscapes of practice : boundaries, identity, and knowledgeability in practice-based learning. New York, NY: Routledge; 2015.

[19] Marshall M, Gordon F. Exploring the role of the interprofessional mentor. J Interprof Care. 8 Apr. 2010;24(4):362–374, DOI:10.3109/13561820903275001 20377398

[20] American medical association: accelerating change in medical education initiative. 2013; http://www.ama-assn.org/sub/accelerating-change/index.shtml.

[21] Gonzalo JD, Lucey C, Wolpaw T, et al. Value-added clinical systems learning roles for medical students that transform education and health: a guide for building partnerships between medical schools and health systems. Acad Med. 2017;92(5):602–607. 27580433 10.1097/ACM.0000000000001346

[22] Creswell JW. Editorial: mapping the field of mixed methods research. J Mix Methods Res. 2009;3(2):95–108.

[23] Braun V, Clarke V 2006. Using thematic analysis in psychology. Qual Res Psychol 3 (2):77–101. DOI:10.1191/1478088706qp063oa

[24] Gonzalo JD, Wolpaw D, Graaf D, et al. Educating patient-centered, systems-aware physicians: a qualitative analysis of medical student perceptions of value-added clinical systems learning roles. BMC Med Educ. 2018;18(1):248. 30384850 10.1186/s12909-018-1345-5 PMC6211412

[25] Lutfiyya MN, Brandt BF, Cerra F. Reflections from the intersection of health professions education and clinical practice: the state of the science of interprofessional education and collaborative practice. Acad Med. 2016;91(6):766–771. 26959223 10.1097/ACM.0000000000001139

[26] de Nooijer J, DHJM D, Stalmeijer RE. Applying landscapes of practice principles to the design of interprofessional education. Teaching and Learning in Medicine Published Online. March 31, 2021;1–6. DOI:10.1080/10401334.2021.1904937 33789558

[27] Dornan T, Tan N, Boshuizen H, et al. How and what do medical students learn in clerkships? Experience based learning (ExBL). Adv Health Sci Educ Theory Pract. 2014;19(5):721–749. DOI:10.1007/s10459-014-9501-0 24638146

[28] Skochelak SE, Hawkins RE. AMA Education Consortium. Health systems science. Philadelphia, PA: Elsevier; 2017.

[29] Gonzalo JD, Baxley E, Borkan J, et al. Priority Areas and Potential Solutions for Successful Integration and Sustainment of Health Systems Science in Undergraduate Medical Education. Acad Med. 2017;92(1):63–69. DOI:10.1097/ACM.0000000000001249 27254015

[30] McDermott C, Shank K, Shervinskie C, et al. Developing a professional identity as a change agent early in medical school: the students’ voice. J Gen Intern Med. 2019;34(5):750–753. 30783879 10.1007/s11606-019-04873-3 PMC6502914

[31] Stoddard HA, Borges NJ. A typology of teaching roles and relationships for medical education. Med Teach. 2016;38(3):280–285. 26075952 10.3109/0142159X.2015.1045848

[32] Hammick M, Freeth D, Koppel I, et al. A best evidence systematic review of interprofessional education. BEME Guide No. 9. Med Teach. 2007;29(8):735–751. 18236271 10.1080/01421590701682576

[33] Lapkin S, Levett-Jones T, Gilligan C. A systematic review of the effectiveness of interprofessional education in health professional programs. Nurse Educ Today. 2013;33(2):90–102. 22196075 10.1016/j.nedt.2011.11.006

[34] Cruess RL, Cruess SR, Steinert Y. Amending miller’s pyramid to include professional identity formation. Acad Med. 2016;91(2):180–185. 26332429 10.1097/ACM.0000000000000913

[35] Skochelak SE, Stack SJ. Creating the Medical Schools of the Future. Acad Med, 2017;92(1):16–19. DOI:10.1097/acm.0000000000001160 27008357

[36] Lucas B. Getting the improvement habit. BMJ Qual Saf. 2016;25(6):400–403. 10.1136/bmjqs-2015-005086 PMC4893130 26717991

[37] Gittell JH, Godfrey M, Thistlethwaite J. Interprofessional collaborative practice and relational coordination: improving healthcare through relationships. J Interprof Care. 2013;27(3):210–213. 23082769 10.3109/13561820.2012.730564

[38] Zwarenstein M, Goldman J, Reeves S. Interprofessional Collaboration to Improve Professional Practice and Healthcare Outcomes. Cochrane Database Syst Rev. 2017;6(6). DOI:10.1002/14651858.cd000072.pub3 PMC6481564 28639262

[39] O’Brien BC, Irby DM. Enacting the Carnegie foundation call for reform of medical school and residency. Teach Learn Med. 2013;25(1):S1–8. 24246101 10.1080/10401334.2013.842915

[40] D’Amour D, Oandasan I. Interprofessionality as the field of interprofessional practice and interprofessional education: an emerging concept. J Interprof Care. 2005;19 Suppl 1(sup1):8–20. 16096142 10.1080/13561820500081604

[41] Parsell G, Bligh J. Interprofessional learning. Postgrad Med J. 1998;74(868):89–95. 9616489 10.1136/pgmj.74.868.89 PMC2360816

[42] Cruess RL, Cruess SR, Steinert Y. Medicine as a community of practice. Acad Med. 2018;93(2):185–191. 28746073 10.1097/ACM.0000000000001826

